# Efficacy of partial spraying of SumiShield, Fludora Fusion and Actellic against wild populations of *Anopheles gambiae* s.l. in experimental huts in Tiassalé, Côte d'Ivoire

**DOI:** 10.1038/s41598-023-38583-y

**Published:** 2023-07-13

**Authors:** Joseph Chabi, Aklilu Seyoum, Constant V. A. Edi, Bernard Loukou Kouassi, Yemane Yihdego, Richard Oxborough, Constant G. N. Gbalegba, Ben Johns, Sameer Desale, Seth R. Irish, John E. Gimnig, Jenny S. Carlson, Melissa Yoshimizu, Jennifer S. Armistead, Allison Belemvire, Lilia Gerberg, Kristen George, Matthew Kirby

**Affiliations:** 1 U.S. President’s Malaria Initiative VectorLink Project, Washington, DC USA; 2 Swiss Center of Scientific Research in Côte d’Ivoire, Abidjan, Côte d’Ivoire; 3 U.S. President’s Malaria Initiative VectorLink Project, Abidjan, Côte d’Ivoire; 4 National Malaria Control Programme, Abidjan, Côte d’Ivoire; 5 Entomology Branch, U.S. President’s Malaria Initiative, U.S. Centers for Disease Control and Prevention, Atlanta, GA USA; 6 U.S. President’s Malaria Initiative, USAID, Washington, DC USA

**Keywords:** Diseases, Medical research

## Abstract

From August 2020 to June 2021, we assessed the efficacy of SumiShield 50WG (clothianidin), Fludora Fusion 56.25WP-SB (mixture of clothianidin and deltamethrin) and Actellic 300CS (pirimiphos-methyl) in experimental huts when partially sprayed against wild, free-flying populations of *Anopheles gambiae* s.l. in Tiassalé, Côte d'Ivoire. A one-month baseline period of mosquito collections was conducted to determine mosquito density and resting behavior in unsprayed huts, after which two treatments of partial indoor residual spraying (IRS) were tested: spraying only the top half of walls + ceilings or only the bottom half of walls + ceilings. These were compared to fully sprayed applications using the three IRS insecticide formulations, during twenty nights per month of collection for nine consecutive months. Mortality was assessed at the time of collection, and after a 24 h holding period (Actellic) or up to 120 h (SumiShield and Fludora Fusion). Unsprayed huts were used as a negative control. The efficacy of each partially sprayed treatment of each insecticide was compared monthly to the fully sprayed huts over the study period with a non-inferiority margin set at 10%. The residual efficacy of each insecticide sprayed was also monitored. A total of 2197 *Anopheles gambiae* s.l. were collected during the baseline and 17,835 during the 9-month period after spraying. During baseline, 42.6% were collected on the bottom half versus 24.3% collected on the top half of the walls, and 33.1% on the ceilings. Over the nine-month post treatment period, 73.5% were collected on the bottom half of the wall, 11.6% collected on the top half and 14.8% on the ceilings. For Actellic, the mean mortality over the nine-month period was 88.5% [87.7, 89.3] for fully sprayed huts, 88.3% [85.1, 91.4] for bottom half + ceiling sprayed walls and 80.8% [74.5, 87.1] for the top half + ceiling sprayed huts. For Fludora Fusion an overall mean mortality of 85.6% [81.5, 89.7] was recorded for fully sprayed huts, 83.7% [82.9, 84.5] for bottom half + ceiling sprayed huts and 81.3% [79.6, 83.0] for the top half + ceiling sprayed huts. For SumiShield, the overall mean mortality was 86.7% [85.3, 88.1] for fully sprayed huts, 85.6% [85.4, 85.8] for the bottom half + ceiling sprayed huts and 76.9% [76.6, 77.3] for the top half + ceiling sprayed huts. For Fludora Fusion, both iterations of partial IRS were non-inferior to full spraying. However, for SumiShield and Actellic, this was true only for the huts with the bottom half + ceiling, reflecting the resting site preference of the local vectors. The results of this study suggest that partial spraying may be a way to reduce the cost of IRS without substantially compromising IRS efficacy.

## Introduction

Indoor residual spraying (IRS) of insecticides has contributed to significant reductions in malaria burden in a range of settings. Between 2000 and 2015, malaria prevalence declined by half and the incidence of clinical disease declined by 40%. Modeling indicated that 11% of the reduction in parasite prevalence was due to IRS^1^, even though IRS coverage in the African region never exceeded 11%^2^.

Globally, the population protected by IRS declined from a peak of 5% in 2010 to 2% in 2019, with decreases seen across all World Health Organization (WHO) regions, apart from the Eastern Mediterranean Region^3^. This decline is concurrent with the switch from relatively inexpensive pyrethroid insecticides to more expensive alternatives to mitigate mosquito resistance. Pyrethroid insecticides were initially replaced with bendiocarb (carbamate), subsequently with pirimiphos-methyl (Actellic, organophosphate) and more recently with clothianidin-based insecticides (SumiShield, and the deltamethrin and clothianidin mixed product Fludora Fusion) in most countries where resistance to any of the insecticides was reported^2^.

The costs of insecticide procurement and IRS implementation remain a challenge to global efforts to reduce malaria. Identifying cost-saving approaches to IRS that are effective and can be widely implemented is imperative to ensuring the sustainability of this proven intervention. The current practice recommended by WHO is for all interior wall, eave and ceiling surfaces to be sprayed in all eligible structures^4^. An alternative approach could be to reduce the surface area sprayed by targeting preferred mosquito resting areas in houses, such as by only spraying the top or bottom half of the walls, in addition to spraying the ceiling or roof (if a ceiling is absent). This approach could potentially reduce the quantity of insecticide needed by 25–50%, reduce operational costs by reducing the time needed to spray a structure, and result in substantial cost savings that could enable an increase in the geographic coverage of IRS. Results from a recent vector behavior study in southern Ghana suggested that the resting preference of vectors depends on the type of roofing material: where the roofing material is thatch and/or there is a ceiling, higher numbers of mosquitoes were collected on the upper wall and ceiling or roof than on the lower part of the wall. In contrast, mosquitoes preferred resting on the lower part of the wall when the roofing was metal with no ceiling^5^. Far fewer mosquitoes were collected on metal roofs in houses without ceilings^6^. In Kenya, Mutinga et al. observed that *Anopheles gambiae* s.l. appeared to prefer resting on the lower section of wall where there was less ambient light^7^. Mosquito resting behavior, if consistent in a given setting or for a given species, has the potential to be exploited through selective IRS application.

In an experimental hut study conducted in Ghana in 2018 using Actellic 300 CS, different partial spraying treatments were compared with a full spray treatment and a negative (no spray) control. Provided ceilings were sprayed, partial spraying of hut walls (either upper or lower half) resulted in a similar level of mortality of wild, free-flying mosquitoes as in fully sprayed huts^6^. A follow-on pilot study to determine the feasibility and cost of a partial IRS campaign and its impact on vector populations under natural field conditions in three IRS communities in northern Ghana was completed in 2019. In that study, the top half of walls and all ceilings were sprayed with Actellic 300 CS. The results showed no significant differences in key entomological indices such as biting rate, parity and indoor resting density of *An. gambiae* s.l. collected from the partial spray IRS communities compared to the full spray IRS communities^6^.

To explore whether partial spraying would be as efficacious as full spraying with currently used IRS insecticides, we conducted an experimental hut study in Côte d’Ivoire using three insecticides: Actellic, SumiShield and Fludora Fusion.

## Results

### Insecticide susceptibility of wild collected *An. gambiae* s.l.

Two rounds of susceptibility tests were conducted to confirm the resistance status of the *An. gambiae* s.l. population of the study site. Resistance was observed to deltamethrin with an average mortality of 11.7% (12.1% and 11.3%) for both test rounds conducted, while susceptibility to pirimiphos-methyl was recorded with an average mortality of 98.5% (97.5% and 99.0%). Susceptibility was observed to clothianidin for both tests with more than 98% (100% and 99%) mortality after 120 h post exposure.

### Baseline mosquito collection

**Figure 1:**
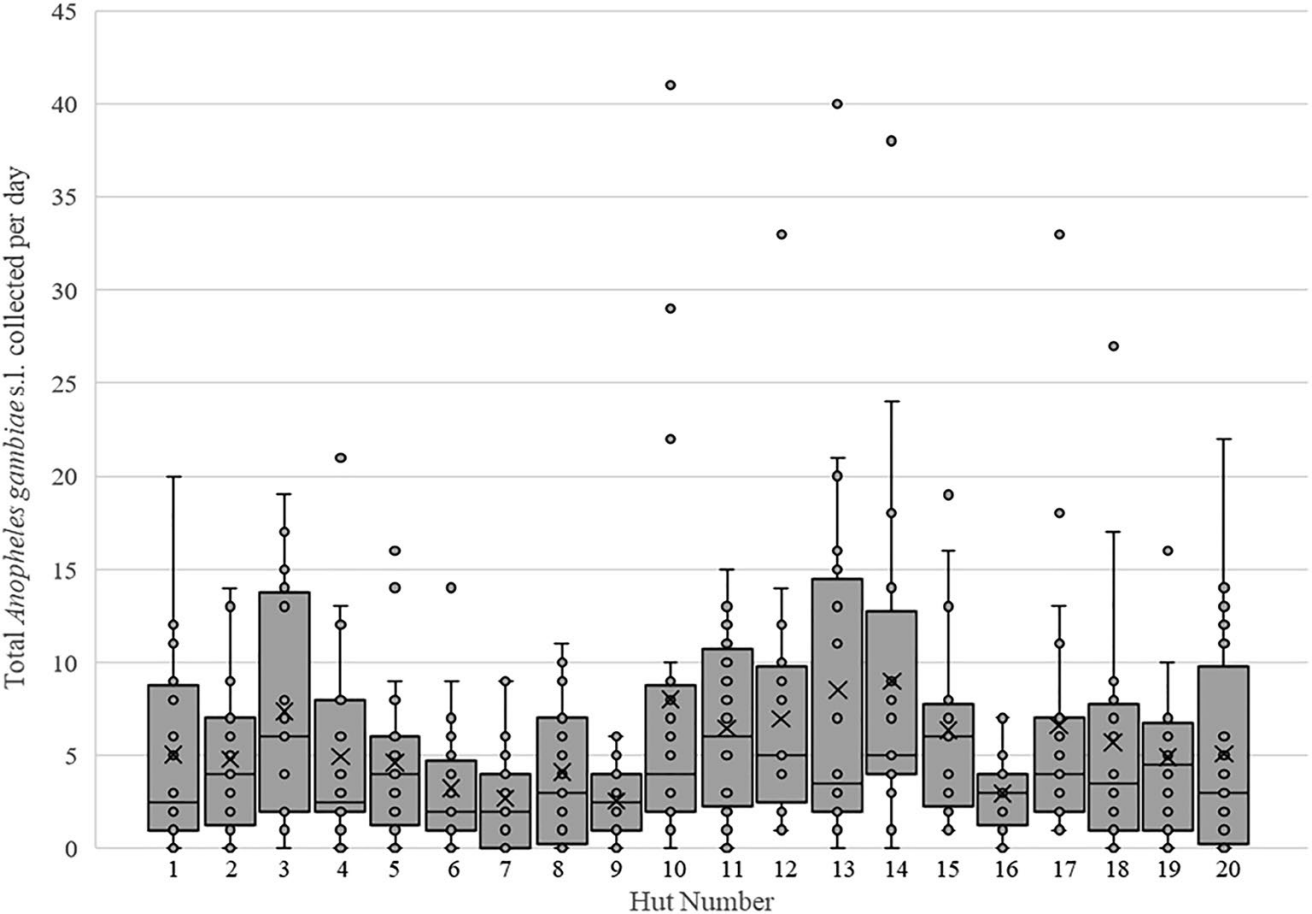
Baseline (pre-spray) collections of *An. gambiae* s.l. Box and whisker plots showing median (horizontal bars) and mean (X symbols) number of mosquitoes collected per hut over the 20-night baseline collection period.

A total of 4698 mosquitoes were collected from all 20 experimental huts in 20 nights during the baseline collection period. Of these, 228 mosquitoes including 43 *An. gambiae* s.l. were collected inside the untreated net used by the sleepers and were excluded in the mosquito resting position calculation. All mosquitoes were collected alive and remained alive after the 24 h holding period. Of the 4698 mosquitoes collected, 46.8% (n = 2197) were identified as *An. gambiae* s.l., and 49.8% were culicines (n = 2340). *Anopheles pharoensis* (2.6%, n = 123) and *An. funestus* s.l. 0.8% (n = 37) were collected in low numbers. Of the 2197 *An. gambiae* s.l., 1513 were collected in the veranda trap and 641 inside the huts, not counting those inside the net (Table 1 and Fig. 1). Of the *An. gambiae* s.l. found resting on sprayable surfaces, 42.6% (273/641) were found on the bottom half of the walls, 24.3% (156/641) on the top half of the walls, and 33.1% were collected from the ceiling.

**Table 1:** Total mosquitoes collected by resting location during the baseline (pre-spray) collection period.

Species	Hut						Veranda	Total
	Bottom half wall (%)	Top half wall (%)	Ceiling (%)	Total inside hut	Inside net*	Total hut	Total veranda*	
*An. gambiae* s.l	273 (42.6)	156 (24.3)	212 (33.1)	641	43	684	1513	2197
Total mosquitoes	376 (27.5)	679 (49.6)	313 (22.9)	1368	228	1596	3102	4698

*The number of mosquitoes collected inside the untreated nets and veranda trap was removed from the percentage per wall resting position calculation.

Overall, the mean baseline density of all mosquitoes collected in the experimental huts was 11.7 mosquitoes per hut per night, and the mean density for *An. gambiae* s.l. was 5.5 per hut per night with a minimum of 2.6 and maximum of 8.5 *An. gambiae* s.l. per hut per night (Supp. Data 1). A difference was noted in density between the huts for *An. gambiae* s.l. (*p* < 0.001) (Fig. 1). As a result, analysis of the main trial accounted for within hut correlations.

### Surface calculation and spraying of the huts

The mean sprayable surface area (walls plus ceiling) of the 20 huts used for the trial was 19.9 m^2^. At an application rate of 30 mL/m^2^ using a 1.5 bar CFV the expected insecticide application volume in the fully sprayed huts was 597 mL. For the partially sprayed huts, the mean sprayable surface area was 12.9 m^2^ and the targeted application volume was 387 mL. The volume applied was slightly more than expected: the means were 811 ml and 425 ml of solution used for full and partial sprayed huts respectively (Supp Data 2). The partial huts were sprayed very accurately—application volumes ranged from 0.5% under sprayed to 16% over sprayed.

### Mosquito species composition and density over the post-spray collection period

A total of 25,834 mosquitoes were collected from all 20 experimental huts over the nine months post-spray collection period. Of these, 17,835 (69.0%) were identified as *An. gambiae* s.l. and 7104 (27.5%) were culicines. The remaining mosquitoes were other *Anopheles* species including 818 *An. pharoensis* (3.2%) and 134 *An. funestus* s.l. (0.5%). Of the 17,835 *An. gambiae* s.l., 971 randomly selected (approximately 100 per month) were identified by PCR to species level. *An. coluzzii* represented more than 99% of the total analyzed. Only seven *An. gambiae* s.s. and two hybrids were recorded.

### Overall efficacy of the treatments against *An. gambiae* s.l

Outcomes for replicate huts for each treatment were combined. A total 4,082 *An. gambiae* s.l. were collected (dead or alive) in the Actellic sprayed huts, 6375 in the Fludora Fusion sprayed huts and 6077 in the SumiShield sprayed huts. The two unsprayed huts yielded 1,301 mosquitoes over the collection period. The proportion of *An. gambiae* s.l. collected in the different positions in the unsprayed control huts during the post spraying collection was similar to that of the baseline (49.3% of the total mosquitoes collected on the bottom half). However, higher percentages of mosquitoes were captured from the lower half of the walls in the sprayed huts. Across all huts and treatments, a mean of 73.5% of mosquitoes collected from inside the hut (excluding the net) were caught from the bottom half (Table 2).

**Table 2:** Total number of *An. gambiae* s.l. collected per location over the nine-month post-spray collection period from all 20 huts.

Insecticide	Hut Treatment	Hut						Veranda	Total
		Bottom half wall (%)	Top half wall (%)	Ceiling (%)	Total inside hut	Inside net*	Total hut	Total veranda*	
	Unsprayed	222 (49.3)	112 (24.9)	116 (25.8)	450	24	474	827	1301
Actellic 300 CS	Full spray	519 (96.5)	9 (1.7)	10 (1.9)	538	7	545	849	1394
	BH + C	552 (94.0)	19 (3.2)	16 (2.7)	587	13	600	887	1487
	TH + C	444 (94.9)	17 (3.6)	7 (1.5)	468	13	481	720	1201
Fludora Fusion WP-SB	Full spray	443 (76.9)	43 (7.5)	90 (15.6)	576	18	594	1284	1878
	BH + C	680 (71.2)	121 (12.7)	154 (16.1)	955	27	982	1399	2381
	TH + C	506 (66.1)	119 (15.5)	141 (18.4)	766	22	788	1328	2116
SumiShield 50 WG	Full spray	491 (70.4)	87 (12.5)	119 (17.1)	697	13	710	1021	1728
	BH + C	617 (66.6)	127 (13.7)	183 (19.7)	927	34	961	1329	2293
	TH + C	415 (60.7)	118 (17.3)	151 (22.1)	684	12	696	1360	2056

For mosquitoes collected from inside the huts, the percentage on the bottom half of the walls, top half of the walls or the ceiling are provided in parentheses.

*BH* bottom half, *TH* top half, *C* ceiling.

*The number of mosquitoes collected inside the untreated net and veranda trap was removed from the percentage per wall resting position calculation.

The overall immediate mean mortality observed with Actellic sprayed huts was 81.6%, higher than that of Fludora Fusion (31.5%) and SumiShield (28.9%) sprayed huts – this is unsurprising because the delayed mortality effects of clothianidin are well known. The mean overall delayed mortality by insecticide (24 h Actellic (86.1% [82.7, 88.9]), 120 h Fludora Fusion (83.5% [81.4, 86.0]) and SumiShield (83.3% [81.1, 85.6])) recorded for all treatments was similar (Tables 3 and 4).

**Table 3:** Total number of *An. gambiae* s.l. collected and overall mortality over the nine-month post-spray collection period.

Insecticide	Hut Treatment	# Alive (morning)	# Dead (morning)	%Immediate mortality	Total collected	# Alive (24 h/120 h)	# Total Dead (24 h/120 h)	% Overall mortality
	Unsprayed*	1293	8	0.6	1301	1287	14	1.1
Actellic 300 CS*	Full spray	233	1161	83.3	1394	163	1231	88.3
	BH + C	226	1261	84.8	1487	176	1311	88.2
	TH + C	294	907	75.5	1201	230	971	80.8
	Total	753	3329	81.6	4082	569	3513	86.1
	Unsprayed**	1293	8	0.6	1301	1216	85	6.5
Fludora Fusion WP-SB**	Full spray	1188	690	36.9	1878	260	1618	86.2
	BH + C	1632	749	31.4	2381	390	1991	83.6
	TH + C	1548	568	26.8	2116	405	1711	80.9
	Total	4368	2007	31.5	6375	1055	5320	83.5
SumiShield 50 WG**	Full spray	1171	560	32.3	1731	221	1507	87.1
	BH + C	1592	698	30.4	2290	326	1967	85.9
	TH + C	1559	497	24.2	2056	470	1586	77.1
	Total	4322	1755	28.9	6077	1017	5060	83.3

*BH* bottom half, *TH* top half, *C* ceiling.

*Delayed mortality was recorded at 24 h.

**Delayed mortality was recorded up to 120 h.

**Table 4:** Overall mortality (unadjusted and adjusted) of *An. gambiae* s.l. and risk ratios for partial spraying treatments in reference to full spraying, over the nine-month post-spray collection period.

Insecticide	Hut treatment	% Mortality unadjusted (95% CI)	% Mortality adjusted^1^ (95% CI)	Risk ratio (95% CI)	p-value
	Unsprayed	6.5 (92.0, 94.7)	6.6 (5.7, 7.5)	–	
Actellic 300 CS*	Full spray	88.3 (86.5, 89.9)	88.5 (87.7, 89.3)	Ref	
	BH + C	88.2 (86.4, 89.8)	88.3 (85.1, 91.4)	0.998 (0.961, 1.035)	0.9
	TH + C	80.7 (78.3, 82.9)	80.8 (74.5, 87.1)	0.913 (0.842, 0.985)	0.0174
Fludora fusion WP-SB**	Full spray	85.8 (84.2, 87.4)	85.6 (81.5, 89.7)	Ref	
	BH + C	83.8 (82.2, 85.2)	83.7 (82.9, 84.5)	0.978 (0.931, 1.026)	0.3697
	TH + C	80.8 (79.1, 82.5)	81.3 (79.6, 83.0)	0.950 (0.900, 0.999)	0.0468
SumiShield 50 WG**	Full spray	87.0 (85.3, 88.6)	86.7 (85.3, 88.1)	Ref	
	BH + C	85.8 (84.3, 87.2)	85.6 (85.4, 85.8)	0.987 (0.971, 1.003)	0.12
	TH + C	77.0 (75.1, 78.8)	76.9 (76.6, 77.3)	0.887 (0.872, 0.903)	< 0.001

*BH* bottom half, *TH* top half, *C* ceiling.

*Mortality at 24 h after collection.

**Mortality at 120 h after collection.

^1^Generalized linear model with repeated measures for hut, adjusting for time since spray date, in months.

### Comparison of efficacy between full and partial spraying

**Figure 2:**
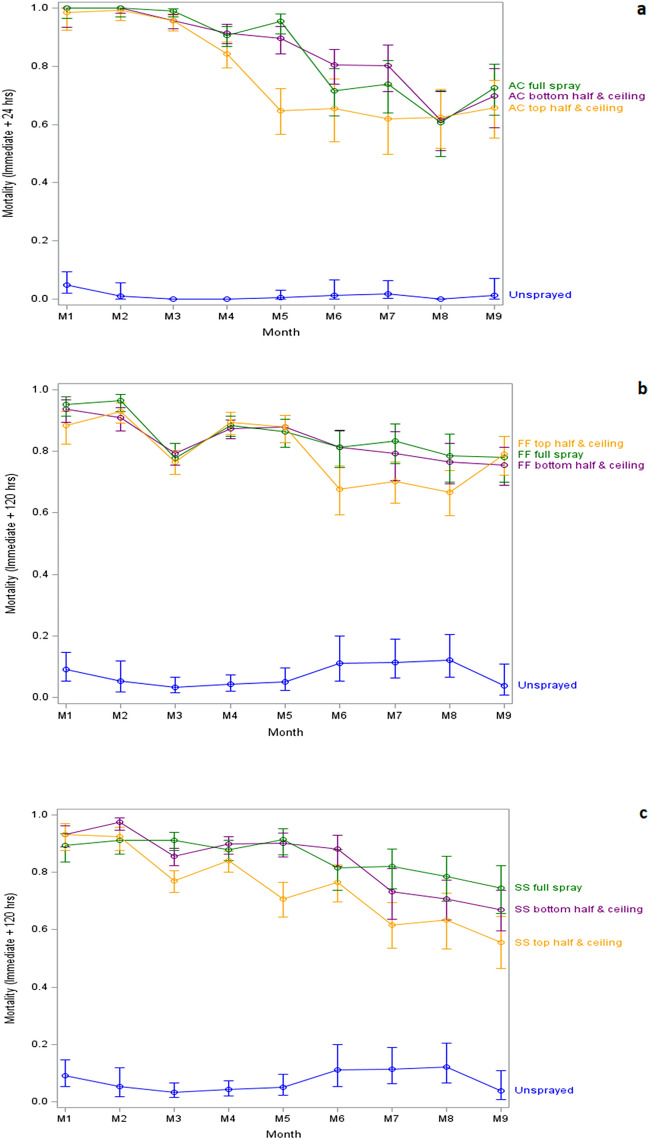
Mean proportion mortality of free-flying *An. gambiae* s.l. in partially or fully sprayed experimental huts up to nine months post-spraying. All percentage mortalities include immediate + 24 h mortality for AC and immediate + 120 h for FF and SS.

Mortality of wild free-flying *An. gambiae* s.l. in huts sprayed with Actellic over the entire post-spray period was 88.5% [87.7, 89.3] for fully sprayed huts, 88.3% [85.1, 91.4] for bottom half + ceiling sprayed walls and 80.8% [74.5, 87.1] for the top half + ceiling sprayed huts (Table 3). Mortality rates were not significantly different between fully sprayed huts and the bottom half + ceiling (RR = 0.99, *p* > 0.91) when the nine-month collection data was pooled, while the fully sprayed hut mortality was higher than that of the top half + ceiling (RR = 0.91 *p* = 0.0174 when compared with full sprayed huts). When comparisons were made for each of the nine months individually no significant differences were observed except in the fifth month when there was significantly higher mortality in the fully sprayed huts compared to the top + ceiling sprayed huts (RR = 0.68, *p* = 0.0008) Fig. 2a, Supp Data 3).

Fludora Fusion efficacy, expressed by monthly mortality up to 120 h post-exposure is presented in Fig. 2b. The overall mean mortality was 85.6% [81.5, 89.7] for the fully sprayed huts, 83.7% [82.9, 84.5] for bottom half + ceiling sprayed huts and 81.3% [79.6, 83.0] for the top half + ceiling sprayed huts. No significant differences were observed for the overall mortality results pooled across the nine months of collection between the fully sprayed huts and bottom half + ceiling sprayed huts (RR = 0.98, p = 0.37). Mortality in top half + ceiling was significantly lower than fully sprayed (RR = 0.95, p = 0.0.0468). While doing monthly comparisons, bottom half + ceiling was significantly lower than fully sprayed in month 2 only, and top half + ceiling was significantly lower in month 2, 6, 7, and 8. (Supp Data 4).

The monthly 120 h mortality in the SumiShield treated huts is presented in Fig. 2c. The overall mean mortality (all months combined) was 86.7% [85.3, 88.1] for fully sprayed huts, 85.6% [85.4, 85.8] for the bottom half + ceiling sprayed huts and 76.9% [76.6, 77.3] for the top half + ceiling sprayed huts. When pooled across the nine months of observation, no difference in mortality was observed between the SumiShield fully sprayed huts and the bottom half + ceiling sprayed huts (RR = 0.99, p = 0.12). However, the mortality of mosquitoes collected in the top half + ceiling sprayed huts was significantly lower than the fully sprayed huts (RR = 0.89, p =  < 0.0001). In monthly analysis, mortality was significantly higher for bottom half + ceiling compared to fully sprayed in month 2 and 6, and it was significantly lower in month 3, 7, 8, and 9. For top half + ceiling mortality was significantly lower than fully sprayed in month 1, 3, and 5 through 9 (Fig. 2c) (Supp Data 5).

**Figure 3:**
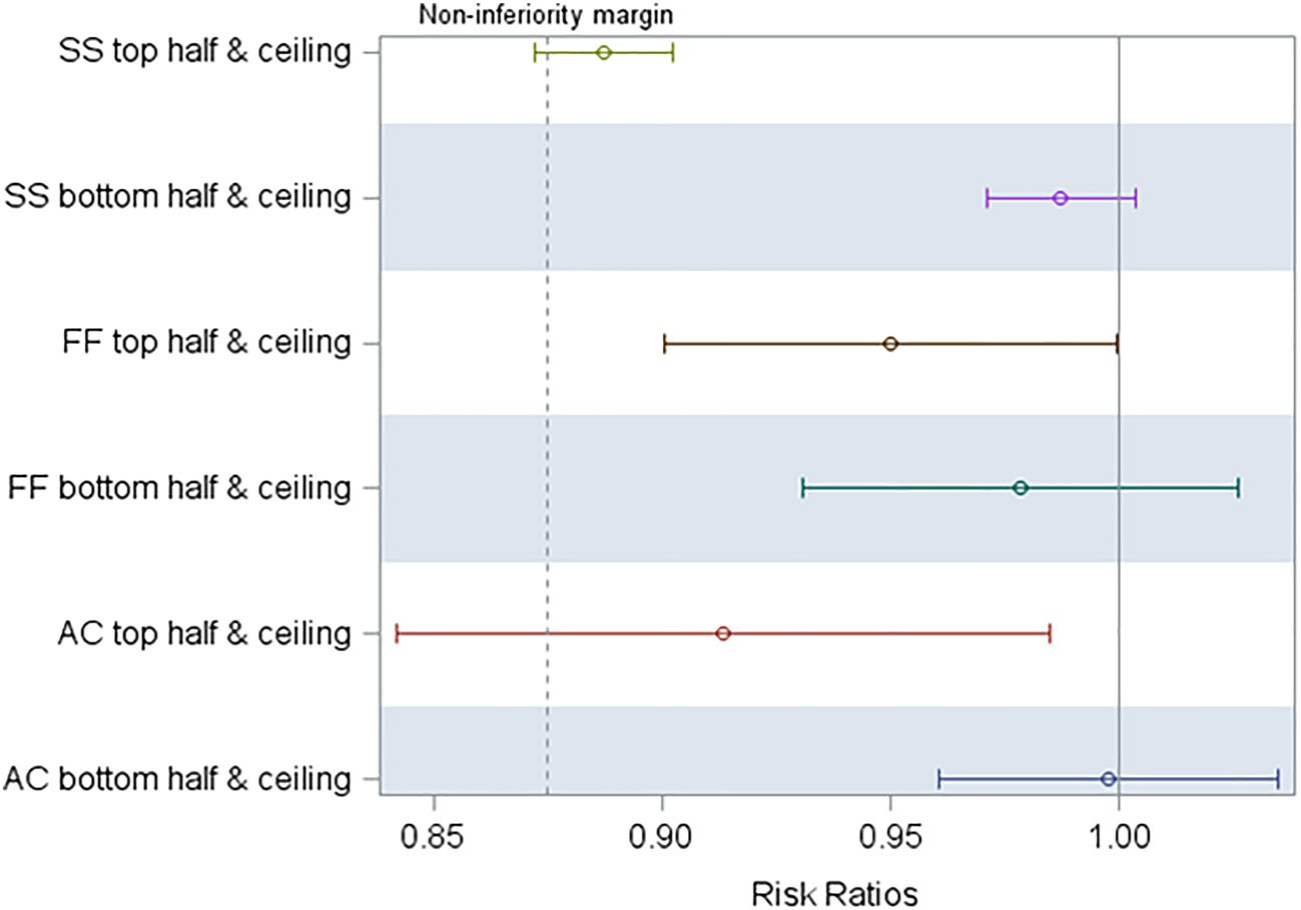
Adjusted risk ratios and 95% confidence interval for partially sprayed treatments compared to fully sprayed treatments for SumiShield (SS), Fludora Fusion (FF), and Actellic (AC).

When comparing the efficacy of partially sprayed treatments to full sprayed treatments, the bottom half + ceiling treatments were non-inferior to the full spraying for all insecticides. The same was true for the top half + ceiling treatment using Fludora Fusion. The lower bound of the confidence interval of the adjusted risk ratio for the mortality induced by the top half + ceiling partial IRS using SumiShield and Actellic overlapped the predetermined non-inferiority margin, demonstrating that those treatments were not non-inferior to the full sprayed treatments of those insecticides (Fig. 3).

### Residual efficacy of sprayed insecticides

The initial cone bioassays conducted in September 2020 immediately after the spraying of the huts showed 100% mortality at 24 h for all three insecticides using the susceptible Kisumu strain.

Residual efficacy was assessed using cone bioassays and with both Kisumu and wild *An. gambiae* s.l. Tiassalé. The results were broadly similar for both test systems and there were no obvious differences between the sprayed surfaces in fully sprayed huts compared to partially sprayed huts. The residual efficacy (> 80% mortality) of SumiShield sprayed on the cement walls was approximately nine months for Kisumu (although there was only one month of data recorded below the 80% threshold) and at least nine months for Tiassalé (> 80% mortality for nine consecutive months). Mortality on walls and ceilings in Fludora Fusion-sprayed huts was > 80% for both test systems throughout the study period, showing that the insecticide could last on the surfaces for more than nine months. The residual efficacy of Actellic was seven months on the sprayed walls (Kisumu and Tiassalé) and eight months on the plywood ceilings (Kisumu only) (Supp Datas 6 and 7).

## Discussion and conclusions

IRS is one of two core vector control interventions recommended by the WHO for malaria control. It has been proven effective and has contributed to considerable decreases in malaria burden in endemic countries where it has been implemented^3,8,9^. However, despite considerable global investments for malaria control, the cost of implementing IRS remains prohibitive for many countries. New cost-saving but comparably effective approaches to implementation are needed to maintain this critical vector control tool. Here, we provide further proof-of-principle evidence for partial IRS (spraying only the top or bottom half of walls, and ceilings) by assessing the efficacy of three IRS insecticide formulations in experimental huts in Côte d’Ivoire.

Mosquito mortality in partially sprayed huts in which the bottom half of walls and the ceiling were sprayed was comparable to that of fully sprayed huts for all three insecticides (Actellic, Fludora Fusion, and SumiShield), suggesting that implementation of partial spraying for IRS may result in similar effectiveness to full spraying. In contrast, the non-inferiority of partial spraying in which the top half of walls and ceiling were sprayed was only demonstrated for Fludora Fusion. Most indoor resting mosquitoes were collected from the bottom half of walls both pre- and post-spraying, which may explain the non-inferiority of this partial spraying iteration for all three insecticides. Nevertheless, mortality was still high in the partially sprayed huts in which the upper half plus ceilings were sprayed. This suggests that mosquitoes may shift resting locations throughout the night while indoors and cannot discriminate between treated and untreated surfaces, nor are repelled or actively move away from treated surfaces. This holds true even for Fludora Fusion, despite the presence of deltamethrin in the mixture, which is a pyrethroid with excito-repellent properties.

Trends in mosquito mortality observed for all three insecticides in this study were similar to those reported in a previous trial in Ghana using Actellic 300 CS. However, in Ghana, mosquitoes were observed to prefer resting on the ceiling rather than the walls of the huts^6^. This may represent differences in resting behavior between members of the *An. gambiae* s.l. complex, as *An. gambiae* s.s. was the primary species collected in Ghana, while *An. coluzzii* was the predominant species within the study site in Côte d'Ivoire. There are no previous reports specifically describing resting habit differences between *An. coluzzii* and *An. gambiae* s.s., or *An. arabiensis*. Also, the differences in resting habits may not reflect species differences but rather might be associated with environmental conditions and microhabitats within the houses as mosquitoes may seek resting locations with optimal temperature and humidity. Msugupukulya et al.reported that 40% of vectors in Tanzania, namely *An. arabiensis* and *An. funestus* s.l., were found resting in the lower part of houses with metal-roofs while a similar proportion were found resting just under the roof in grass-thatched houses^10^.

These observations highlight the need to determine vector resting behavior, ideally at multiple time points, to understand any movement across wall surfaces during the night, as well as the impact of housing type prior to planning and implementing partial IRS^11,12^. If local vectors prefer to rest on the bottom half of walls and are not observed to move during the night, partial spraying must target the bottom half of walls. Spraying only the bottom half of walls, whilst reducing costs by decreasing insecticide used, is likely to be less cost-effective than spraying only the top half of walls. The potential time and cost savings in terms of house preparation, including moving of house furniture and goods before spraying observed when spraying only the upper half of walls during the trial in Ghana, would likely be lost when spraying only the bottom half of walls^6^.

Of all three insecticides tested, only Fludora Fusion performed similarly in both iterations of partially sprayed huts compared to fully sprayed huts and demonstrated the longest residual efficacy against both mosquito strains, which may present a slight advantage over the other two products. However, Actellic and SumiShield did perform as well when applied to bottom half of wall plus ceiling compared to full spraying. This suggests that all three insecticides may be suitable for partial IRS, but insecticide selection should still be driven by local data on insecticide resistance patterns and residual efficacy.

Various approaches to partial spraying for malaria vector control have been evaluated previously, including in Mexico against *An. albimanus*^13^, in the Philippines with *An. flavirostris*^14^, in Lebanon against *An. sacharovi*^15^ and in Ghana against *An. gambiae* s.l.^6^. All these trials reported promising results, but partial IRS has yet to be adopted at scale. The current study provides further proof-of-principle evidence on the efficacy of partial spraying and highlights the need for a larger randomized control trial to confirm the effectiveness of partial IRS versus full IRS in terms of epidemiological impact to support decision making for wider implementation. Furthermore, a detailed cost analysis of partial spraying will be needed before wider implementation of this approach.

This study had a certain number of limitations that should be considered when interpreting the results. First, mosquito resting behavior was measured at only a single time point (between 5 and 6am) during this study, and may not be completely representative of the resting behavior of local populations of *An. gambiae* s.l. The high mortality observed in all insecticide treatments, including both partial spray treatments, suggests that mosquitoes may move up and down the walls during the night and therefore contact treated surfaces even if a hut is only partially sprayed. Secondly, the estimated insecticide dose exceeded the target dose in most huts though it was still within the acceptable range of ± 50%^16^ and it is unlikely that this drastically affected the outcomes of the study. It should also be noted that the full spraying exceeded the target dose by a greater amount than both partial spray treatments for all insecticides, and yet non-inferiority was still demonstrated for the bottom half + ceiling treatment.

In conclusion, partial IRS (bottom half of wall + ceiling) is not substantially less effective than full spraying in this setting, when considering entomological indicators only, but additional studies are needed to explore the epidemiological impact. This study also demonstrated the need for initial assessment of the vector resting behavior before partial IRS is implemented at scale. Partial IRS represents an opportunity to greatly reduce the overall costs of IRS, allowing for expansion of this highly effective vector control tool.

## Methods

### Study site

**Figure 4:**
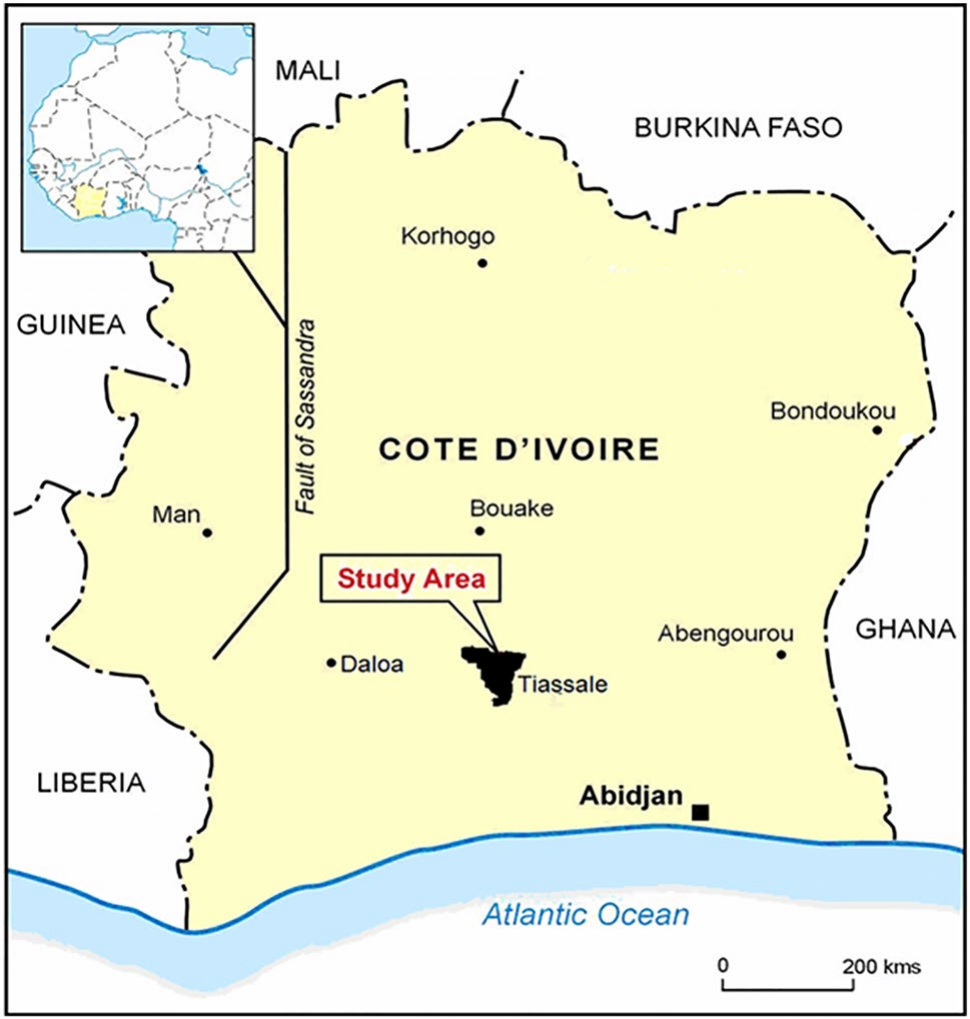
Study site in the district of Tiassalé, Cote d’Ivoire.

The trial was conducted in experimental huts located in Tiassalé District (5.889542, − 4.8287673), southern Côte d’Ivoire (Fig. 4) where *An. coluzzii* is the major malaria vector^17^. Results from WHO tube tests conducted by the Swiss Center for Scientific Research (CSRS) have previously shown that the vector in this area is resistant to all pyrethroids, with a high frequency (79%) of the west African knockdown resistance allele L1014F (*kdr-w*)^17^. Mosquito densities are high throughout the year due to year-round irrigation of rice fields in the area^18^.

### Design of the experimental huts

Twenty experimental huts based on the West Africa design (described in WHO guidelines^19^) were used. Each hut is about 2.0 m wide by 3.5 m long including the veranda trap, and 2.0 m high at the front side of the hut. Huts are made from concrete bricks with a corrugated iron roof and a plywood ceiling. The veranda measuring 2.0 m long, 1.5 m wide and 1.5 m high is made of thick polypropylene tarpaulin and untreated screening mesh. Each hut stands on a concrete base surrounded by a water-filled moat to exclude ants that would otherwise carry off dead mosquitoes. Mosquitoes enter through four modified slits located on three sides (front and two sides) of the hut. The slits are designed to allow for entry but impede exit while the veranda projecting from the fourth side is designed to capture mosquitoes that attempt to exit^20^.

### Susceptibility testing

WHO susceptibility tests were conducted during the baseline collection period in September 2020 and after the spraying of the huts in October 2020 to assess the susceptibility status of the local wild *An. gambiae* s.l. population against the insecticides to be sprayed: clothianidin (2%), deltamethrin (0.05%) and pirimiphos-methyl (0.25%). Deltamethrin and pirimiphos-methyl impregnated papers were obtained from Universiti Sains Malaysia while the clothianidin-based papers were prepared locally using the formulated SumiShield product. The papers were impregnated using 264 mg of SumiShield diluted in 20 ml of distilled water. Two milliliters of the solution were used to impregnate each 12 cm × 15 cm Whatman grade 1 filter paper^21^. For the tests, larvae were collected around the experimental huts and raised to adults. Approximately 100 females, 3–5 days old, were exposed to each insecticide to confirm the susceptibility status to the test insecticides, according to WHO protocols^22^.

### Hut treatments

Two configurations of partial IRS (top half of walls + ceiling, bottom half of walls + ceiling) were compared with a negative, unsprayed control and fully sprayed positive control huts with three different insecticide formulations. The treatment arms included:

#### Negative control

- Unsprayed huts

#### Positive controls (fully sprayed)

- SumiShield at 300 mg active ingredient (a.i.)/m^2^ clothianidin
- Fludora Fusion at a mixture of 200 mg a.i./m^2^ of clothianidin and 25 mg a.i./m^2^ deltamethrin
- Actellic at 1 g a.i. /m^2^ pirimiphos-methyl

#### Experimental

- SumiShield at 300 mg a.i/m^2^ clothianidin (top half of walls and ceiling sprayed)
- Fludora Fusion at a mixture of 200 mg a.i./m^2^ of clothianidin and 25 mg a.i./m^2^ deltamethrin (top half of walls and ceiling sprayed)
- Actellic at 1 g a.i./m^2^ (top half of walls and ceiling sprayed)
- SumiShield at 300 mg a.i/m^2^ (bottom half of walls and ceiling sprayed)
- Fludora Fusion at a mixture of 200 mg a.i./m^2^ of clothianidin and 25 mg a.i./m^2^ deltamethrin (bottom half of walls and ceiling sprayed)
- Actellic at 1 g a.i./m^2^ (bottom half of walls and ceiling sprayed)

Two huts were randomly assigned to each of the 10 different treatments. For the partial spray treatments, either the ceiling and the top half of the walls, (i.e., measured starting from the ceiling to one meter down the walls) or the ceiling and the bottom half of the walls, (i.e., measured starting from the floor up to one meter above the floor) were sprayed. A demarcation line was drawn with a permanent marker in each hut allocated to partial spraying to indicate the area to be sprayed to guide the spray operators. The insecticide was mixed in 7.5L of tap water in the Goizper pumps (Goizper Group, IK, Spain) and shaken for complete and homogenous dispersion following the manufacturer’s instructions for preparing the spray solutions. All six huts sharing a common insecticide were sprayed from the same tank mix and by the same spray operator.

Spraying was conducted on September 12, 2020, following standard procedures^4^, using the Goizper pumps fitted with a controlled flow valve. The volume of the insecticide solution was measured before and after spraying to quantify the amount of insecticide solution applied. The sprayable surface area of all walls and ceilings of huts was measured to estimate the dosage of insecticide sprayed per square meter.

### Mosquito collections

Residents in the community were hired to sleep in the huts and were trained to collect the mosquitoes each morning before opening the doors and leaving the huts. They were provided with new intact untreated mosquito nets and were rotated nightly between huts so that each sleeper occupied each hut for one night during a complete rotation of 20 nights, to correct for any differences in attractiveness. Sleepers entered the huts at 8:00 pm and remained inside until 6:00 am to collect the mosquitoes that had entered the huts during the night.

Baseline collections were carried out for 20 nights from August 20th to September 10th, 2020 (one month prior to spraying), in part to determine if there were natural variations in mosquito densities between huts, and in part to record resting position on the wall (top half, bottom half or ceiling) which could contribute to the data interpretation for the main study. The spraying was followed by nine consecutive monthly collections, each consisting of 20-collection days from all huts. The treatment arms were not rotated due to logistical constraints of rotating IRS substrates.

All mosquitoes, dead or alive, were collected every morning between 5:30 am and 6:00 am at each section of the huts and always in the same order: inside the net (after which the veranda cloth was released separating the compartments), then floor, bottom half of the wall, top half of the wall, ceiling, and lastly the veranda. Forceps were used to collect the dead mosquitoes and 5 mL hemolysis tubes covered with cotton wool for live mosquitoes. For both baseline collection and post spray collections, the collectors were guided by the demarcation lines on hut walls to record each section from which mosquitoes were caught. Mosquitoes collected from each section of the hut were put in separate collection bags. The mosquitoes were identified morphologically using dichotomous keys^23^, and scored by location as dead or alive and as blood fed or unfed. All live mosquitoes were kept in disposable cups at the site laboratory and fed with sugar solution (10%). Delayed mortality was recorded at 24 h for Actellic and daily for up to 120 h for both SumiShield and Fludora Fusion.

### Cone bioassays

Cone bioassays were conducted the day after spraying to confirm the quality of the spraying, and subsequently every month for residual efficacy monitoring, according to WHO guidelines^24^. In the fully sprayed huts, one cone was positioned on the lower half of the wall, one on the upper half of a different wall and one on the sprayed ceiling. In the partial sprayed huts, two cones were placed at the same height on the sprayed portion of the wall (the sprayed bottom or top half), but on different sides of the hut, and one cone was positioned on the ceiling. The location of each cone was marked and used repeatedly over the testing period. About ten 3–5-day-old female *An. gambiae* s.s. Kisumu strain (fully susceptible) were introduced per cone and exposed for 30 min. Similarly, about ten 3–5-day-old wild female *An. gambiae* s.l. mosquitoes that had emerged from local larval collections were also tested every month. Thus, approximately 600 *An. gambiae* Kisumu strain and 600 wild mosquitoes were used for each monthly round of cone bioassays. Knock down was recorded after 60 min and mortality scored at 24 h for Actellic, or daily up to 120 h after exposure for Fludora Fusion and SumiShield. Similarly, control hut delayed mortality was considered at 24 h for Actellic and up to 120 h for Fludora Fusion and SumiShield.

### Molecular identification

All *An. gambiae* s.l. mosquitoes collected in the huts during the trial were preserved individually in Eppendorf tubes containing silica gel. About 100 specimens were randomly selected each month and analyzed for species identification using polymerase chain rection (PCR) methods. The DNA of each individual mosquito was extracted using the protocol designed by Collins et al.^25^. *Anopheles gambiae* complex species were identified as *An. gambiae* s.s., *An. coluzzii,* or hybrids of the two species, following the Short-Interspersed Element protocol described by Santolamazza et al*.*^26^.

### Descriptive analyses

At baseline (pre-spraying), mosquito counts were disaggregated by location (hut vs. veranda), feeding status (blood fed vs. unfed), and position inside hut (top/bottom wall, ceiling, inside net). For the main trial (post-spraying), mosquito counts (alive, dead, and total) were disaggregated by insecticide type (Control, Actellic, SumiShield, and Fludora Fusion) and treatment type (fully vs. partially sprayed (top half or bottom half)). The total number dead at 24 h for Actellic and 120 h for SumiShield and Fludora Fusion was then divided by the number of mosquitoes captured to determine the mortality by treatment and time period.

### Sample size and power calculations

Mosquito collections in the experimental huts were carried out for 20 consecutive nights every month for nine consecutive months.

#### Assumptions

- A mean of five female *Anopheles* of the same species enters each experimental hut either to rest or feed every day.
- On average, full spraying kills 80% of the mosquitoes that enter the experimental huts.
- An absolute difference of 10% vector mortality is acceptable between the partially sprayed and fully sprayed experimental huts (10% lower effect size in partial sprayed huts as compared to fully sprayed).
- All partially sprayed houses have similar effect size (70% mortality of mosquitoes entering the houses).
- Significance level of 0.05 and equal sample sizes for all treatments.

With an assumed design effect of 1.26 and based on the above assumptions, a total of 1,680 mosquitoes per comparison were required to have 90% power to test non-inferiority of partially sprayed huts with a non-inferiority margin of 10% mosquito mortality compared to fully sprayed huts. Comparisons were made between each treatment that shared a common insecticide, for individual months and for all months combined.

### Statistical analysis

The number of mosquitoes entering the huts, the proportion of mosquitoes that exited by morning (those collected in the veranda), the proportion that were found dead in the morning or died during the holding periods, and the number dying relative to the control were compared between treatment and control arms.

Analysis was performed using generalized estimating equations (GEE) model with repeated measures per hut. The primary outcome i.e., mosquito mortality, was a binary variable with value dead or alive, where dead for Actellic indicated dead at 24 h and for Fludora Fusion and SumiShield indicated dead at 120 h. The model was constructed with the outcome event as mosquito death, with treatment and time post-spraying in months as fixed effects. The treatment groups had a total of ten levels. Three levels (Full Spray, Bottom Half + Ceiling (BH + C), Top Half + Ceiling (TH + C)) each for Actellic, Fludora Fusion, and SumiShield, and control group. The analysis was conducted in SAS version 9.4 using ‘margins’ macro. Hut was set as a subject, within hut correlation type was set as autoregressive of lag one. Risk ratios were calculated for each insecticide comparing BH + C, and TH + C to full spray. Unadjusted proportions and confidence intervals using binomial distribution and adjusted proportions and confidence intervals using the regression model defined above were calculated. The GEE model defined above was also used to model monthly estimates. Proportions, risk ratios, and their confidence intervals are presented in supplemental tables.

Non inferiority margin of 10% was defined assuming 80% mortality in fully sprayed and 70% mortality in partially sprayed, indicating risk ratio of 0.875 (RR = 0.7/0.8 for partially sprayed compared to fully sprayed. Therefore, partial spraying would be deemed non-inferior to full spraying if the lower bound of 95% confidence interval of the risk ratio was greater than 0.875.

### Ethical permission

Ethical approval for this study was obtained from the Institutional Review Board (IRB) of Cote d’Ivoire under the registration number 079–20/MSHP/CNESVS-kp while Abt Associates IRB authorized the study before starting the trial. Informed consent was obtained from all volunteers (sleepers) and mosquito collectors that participated in the study. All methods were performed in accordance with relevant guidelines and regulations.

### Conditions to participate to the study

Sleepers: Only male adults of 18 years or older were selected to sleep in the experimental huts. Adequate security measures were put in place to protect the safety of volunteer sleepers. All volunteers were selected from the study village community. Volunteers live locally and they were remunerated for participating in the study. The risks of malaria were also explained. Volunteers were monitored each day for signs of fever or possible side effects of the insecticide. Volunteers were screened regardless of signs and symptoms using a WHO recommended malaria rapid diagnostic test kit at the time of enrollment, and every four weeks during the trial and four weeks after completion. No confirmed uncomplicated *Plasmodium falciparum* parasitemia case and adverse effect associated with the insecticides sprayed was reported during the trial. Additionally, a COVID-19 mitigation plan was put in place at the hut station including hand washing with liquid soap and water, nose mask and social distancing which resulted in no COVID-19 cases report during the study.

## Supplementary Information

Supplementary Information 1.

Supplementary Information 2.

Supplementary Information 3.

Supplementary Information 4.

Supplementary Information 5.

Supplementary Information 6.

Supplementary Information 7.

## Data Availability

All data generated or analyzed during this study are included in this published article and its supplementary information files. However, it can be made available by the corresponding author upon reasonable request.

## Abbreviations

CDC: Centers for disease control and prevention
CS: Concentrated suspension
IRS: Indoor residual spraying
ITN: Insecticide-treated net
NMCP: National malaria control programme
PCR: Polymerase chain reaction
SINE: Short interspersed element
WG: Wettable granule
WHO: World health organization
WP: Wettable powder
