# Supplementary material for: Efficacy of partial spraying of SumiShield, Fludora Fusion and Actellic against wild populations of Anopheles gambiae s.l. in experimental huts in Tiassalé, Côte d'Ivoire
Source: Sci Rep. 2023 Jul 13;13:11364. doi: 10.1038/s41598-023-38583-y (PMC10344869; doi:10.1038/s41598-023-38583-y)
Supplement: Supplementary file 1 — Supplementary Information 1. [file 41598_2023_38583_MOESM1_ESM.pdf]

Supp data 2: Total and mean mosquitoes and *An. gambiae* s.l. collected per hut during the 20-night baseline collections

Hut #	Total *An. gambiae* s.l.	Mean *An. gambiae* s.l./hut/night	Total all mosquito species	Mean number mosquitoes/hut/night
Hut 1	101	5.1	195	9.8
Hut 2	96	4.8	178	8.9
Hut 3	147	7.4	301	15.1
Hut 4	99	5	188	9.4
Hut 5	92	4.6	186	9.3
Hut 6	65	3.3	168	8.4
Hut 7	54	2.7	141	7.1
Hut 8	82	4.1	179	9
Hut 9	51	2.6	105	5.3
Hut 10	160	8	280	14
Hut 11	129	6.5	274	13.7
Hut 12	139	7	268	13.4
Hut 13	170	8.5	415	20.8
Hut 14	180	9	435	21.8
Hut 15	127	6.4	243	12.2
Hut 16	59	3	149	7.5
Hut 17	132	6.6	245	12.3
Hut 18	114	5.7	264	13.2
Hut 19	98	4.9	239	12
Hut 20	102	5.1	245	12.3
**Total**	**2197**	**5.5**	**4698**	**11.7**
