# Supplementary material for: Efficacy of partial spraying of SumiShield, Fludora Fusion and Actellic against wild populations of Anopheles gambiae s.l. in experimental huts in Tiassalé, Côte d'Ivoire
Source: Sci Rep. 2023 Jul 13;13:11364. doi: 10.1038/s41598-023-38583-y (PMC10344869; doi:10.1038/s41598-023-38583-y)
Supplement: Supplementary file 2 — Supplementary Information 2. [file 41598_2023_38583_MOESM2_ESM.pdf]

Supp data 1: Insecticide dosage per treated hut surfaces as estimated from the difference between the volume of the insecticide solution before and after spraying (BH = bottom half, TH = top half, C = ceiling, a.i. = active ingredient)

Insecticides	Hut #	Huts	Hut Surface area m^2	Hut Sprayed surface m^2	Expected volume of application in ml (30ml/m^2)	Actual volume of application in ml	Difference in ml	Percentage volume applied	Target dose a.i. (g) / m^2	Actual application a.i. (g /m^2)	Difference in dosage a.i. (g/ m^2)
**Actellic 300 CS**	5	Full spray	19.98	19.98	599.4	790	190.6	131.8%	1	1.37	0.37
	15	Full spray	19.63	19.63	588.9	760	171.1	129.0%	1	1.34	0.34
	9	BH+C	19.26	12.52	375.6	400	24.4	106.5%	1	1.06	0.06
	16	BH+C	20.54	13.49	404.7	500	95.3	123.5%	1	1.24	0.24
	7	TH+C	20.1	12.95	388.5	400	11.5	103.0%	1	1.03	0.03
	17	TH+C	20.15	13.2	396	450	54	113.6%	1	1.14	0.14
**Fludora Fusion WP-SB**	13	Full spray	20.11	20.11	603.3	820	216.7	135.9%	0.225	0.32	0.09
	18	Full spray	20.91	20.91	627.3	900	272.7	143.5%	0.225	0.34	0.11
	6	BH+C	20.3	12.99	389.7	410	20.3	105.2%	0.225	0.24	0.01
	12	BH+C	18.28	11.97	359.1	370	10.9	103,0%	0.225	0.23	0.01
	11	TH+C	18.12	11.85	355.5	380	24.5	106.9%	0.225	0.24	0.02
	20	TH+C	21.98	14.21	426.3	450	23.7	105.6%	0.225	0.24	0.01
**SumiShield 50 WG**	2	Full spray	19.84	19.84	595.2	850	254.8	142.8%	0.30	0.44	0.14
	10	Full spray	19.34	19.34	580.2	750	169.8	129.3%	0.30	0.40	0.10
	3	BH+C	19.18	12.51	375.3	480	104.7	127.9%	0.30	0.38	0.08
	19	BH+C	20.74	13.68	410.4	450	39.6	109.6%	0.30	0.33	0.03
	4	TH+C	19.19	12.49	374.7	400	25.3	106.8%	0.30	0.32	0.02
	8	TH+C	19.2	12.49	374.7	390	15.3	104.3%	0.30	0.31	0.01
**Control**	1	Unsprayed	21.45	NA	NA	NA	NA	NA	NA	NA	NA
	14	Unsprayed	19.76	NA	NA	NA	NA	NA	NA	NA	NA
