# Supplementary material for: Efficacy of partial spraying of SumiShield, Fludora Fusion and Actellic against wild populations of Anopheles gambiae s.l. in experimental huts in Tiassalé, Côte d'Ivoire
Source: Sci Rep. 2023 Jul 13;13:11364. doi: 10.1038/s41598-023-38583-y (PMC10344869; doi:10.1038/s41598-023-38583-y)
Supplement: Supplementary file 3 — Supplementary Information 3. [file 41598_2023_38583_MOESM3_ESM.pdf]

Supp data 3: Analysis of fully versus partially sprayed huts per month for Actellic sprayed huts. Comparisons are made across each row with the fully sprayed huts as the reference category for each row. Numbers in parentheses for mortality and risk ratios represent the 95% confidence intervals.

	AC Full Unadjusted % Mortality (95% CI)	AC BH+C Unadjusted % Mortality (95% CI)	Risk Ratio	P value	AC TH+C Unadjusted % Mortality (95% CI)	Risk Ratio	P value
M1	1.00 (0.96, 1.00)	1.00 (0.93, 1.00)	-	-	1.00 (1.00, 1.00)	-	-
M2	1.00 (0.97, 1.00)	1.00 (0.98, 1.00)	-	-	1.00 (1.00, 1.00)	-	-
M3	0.99 (0.97, 1.00)	0.96 (0.93, 0.98)	0.97 (0.92, 1.02)	0.2148	0.96 (0.92, 0.99)	0.97 (0.93, 1.00)	0.0483
M4	0.91 (0.87, 0.94)	0.91 (0.88, 0.94)	1.01 (0.86, 1.15)	0.9091	0.84 (0.79, 0.89)	0.93 (0.87, 0.98)	0.0124
M5	0.95 (0.91, 0.98)	0.90 (0.84, 0.94)	0.94 (0.88, 1.00)	0.0500	0.65 (0.47, 0.83)	0.68 (0.49, 0.87)	0.0008
M6	0.72 (0.63, 0.79)	0.80 (0.74, 0.86)	1.12 (0.92, 1.31)	0.2400	0.65 (0.45, 0.86)	0.91 (0.58, 1.24)	0.6043
M7	0.74 (0.64, 0.82)	0.80 (0.71, 0.87)	1.09 (0.92, 1.25)	0.2986	0.62 (0.55, 0.69)	0.84 (0.68, 1.00)	0.0490
M8	0.61 (0.49, 0.72)	0.61 (0.51, 0.71)	1.01 (0.89, 1.13)	0.8523	0.62 (0.57, 0.68)	1.03 (0.94, 1.12)	0.5268
M9	0.72 (0.63, 0.81)	0.70 (0.59, 0.79)	0.95 (0.80, 1.10)	0.5382	0.65 (0.65, 0.66)	0.90 (0.76, 1.04)	0.1787

- Month 1 and 2, mortality rate in full and bottom half, both were 100%
- AC: Actellic 300 CS; BH: bottom half, TH: top half, C: ceiling; M: month
