# Supplementary material for: Efficacy of partial spraying of SumiShield, Fludora Fusion and Actellic against wild populations of Anopheles gambiae s.l. in experimental huts in Tiassalé, Côte d'Ivoire
Source: Sci Rep. 2023 Jul 13;13:11364. doi: 10.1038/s41598-023-38583-y (PMC10344869; doi:10.1038/s41598-023-38583-y)
Supplement: Supplementary file 4 — Supplementary Information 4. [file 41598_2023_38583_MOESM4_ESM.pdf]

Supp data 4: Analysis of fully versus partially sprayed huts per month for Fludora Fusion sprayed huts. Comparisons are made across each row with the fully sprayed huts as the reference category for each row. Numbers in parentheses for mortality and risk ratios represent the 95% confidence intervals.

	FF Full Unadjusted % Mortality (95% CI)	FF BH+C Unadjusted % Mortality (95% CI)	Risk Ratio	P value	FF TH+C Unadjusted % Mortality (95% CI)	Risk Ratio	P value
M1	0.95 (0.91, 0.98)	0.94 (0.89, 0.97)	0.98 (0.94, 1.03)	0.4781	0.89 (0.89, 0.89)	0.93 (0.92, 0.94)	<.0001
M2	0.97 (0.93, 0.99)	0.91 (0.87, 0.94)	0.94 (0.9, 0.98)	0.0022	0.93 (0.91, 0.95)	0.96 (0.93, 1.00)	0.0441
M3	0.78 (0.73, 0.83)	0.79 (0.76, 0.83)	1.02 (0.94, 1.09)	0.6772	0.76 (0.71, 0.82)	0.98 (0.88, 1.08)	0.7064
M4	0.88 (0.85, 0.91)	0.87 (0.84, 0.90)	0.99 (0.94, 1.04)	0.6203	0.89 (0.82, 0.97)	1.01 (0.93, 1.09)	0.7767
M5	0.86 (0.81, 0.90)	0.88 (0.83, 0.92)	1.01 (0.96, 1.07)	0.6256	0.88 (0.84, 0.91)	1.02 (0.96, 1.08)	0.5861
M6	0.81 (0.75, 0.87)	0.81 (0.75, 0.87)	1.00 (0.89, 1.10)	0.9390	0.68 (0.66, 0.7)	0.83 (0.74, 0.93)	0.0004
M7	0.83 (0.76, 0.89)	0.79 (0.71, 0.86)	-	-	0.7 (0.63, 0.77)	0.84 (0.76, 0.93)	0.0003
M8	0.78 (0.70, 0.86)	0.76 (0.69, 0.83)	0.97 (0.89, 1.01)	0.1351	0.67 (0.62, 0.71)	0.85 (0.78, 0.92)	<.0001
M9	0.78 (0.70, 0.85)	0.76 (0.69, 0.81)	0.96 (0.85, 1.06)	0.4004	0.79 (0.76, 0.81)	1.01 (0.92, 1.09)	0.9018

- Only one hut was available in month 7 for bottom half
- FF: Fludora Fusion WP-SB; BH: bottom half, TH: top half, C: ceiling; M: month
