# Supplementary material for: Efficacy of partial spraying of SumiShield, Fludora Fusion and Actellic against wild populations of Anopheles gambiae s.l. in experimental huts in Tiassalé, Côte d'Ivoire
Source: Sci Rep. 2023 Jul 13;13:11364. doi: 10.1038/s41598-023-38583-y (PMC10344869; doi:10.1038/s41598-023-38583-y)
Supplement: Supplementary file 5 — Supplementary Information 5. [file 41598_2023_38583_MOESM5_ESM.pdf]

Supp data 5: Analysis of fully versus partially sprayed huts per month for SumiShield sprayed huts. Comparisons are made across each row with the fully sprayed huts as the reference category for each row. Numbers in parentheses for mortality and risk ratios represent the 95% confidence intervals.

	SS Full Unadjusted % Mortality (95% CI)	SS BH+C Unadjusted % Mortality (95% CI)	Risk Ratio	P value	SS TH+C Unadjusted % Mortality (95% CI)	Risk Ratio	P value
M1	0.89 (0.84, 0.94)	0.93 (0.89, 0.96)	1.04 (0.99, 1.09)	0.0823	0.93 (0.91, 0.95)	1.04 (1.00, 1.09)	0.0449
M2	0.91 (0.86, 0.95)	0.97 (0.95, 0.99)	1.07 (1.04, 1.10)	<.0001	0.92 (0.90, 0.95)	1.01 (0.97, 1.05)	0.5254
M3	0.91 (0.88, 0.94)	0.85 (0.82, 0.88)	0.94 (0.89, 0.98)	0.0056	0.77 (0.72, 0.81)	0.84 (0.79, 0.89)	<.0001
M4	0.88 (0.84, 0.91)	0.90 (0.86, 0.93)	1.02 (0.96, 1.08)	0.4623	0.84 (0.82, 0.86)	0.96 (0.90, 1.01)	0.1352
M5	0.91 (0.86, 0.95)	0.90 (0.85, 0.94)	0.98 (0.95, 1.02)	0.3692	0.70 (0.66, 0.75)	0.77 (0.72, 0.82)	<.0001
M6	0.82 (0.74, 0.88)	0.88 (0.82, 0.93)	1.08 (1.07, 1.10)	<.0001	0.76 (0.76, 0.76)	0.94 (0.92, 0.95)	<.0001
M7	0.82 (0.74, 0.88)	0.73 (0.64, 0.81)	0.89 (0.86, 0.93)	<.0001	0.62 (0.50, 0.74)	0.75 (0.60, 0.90)	0.0013
M8	0.78 (0.70, 0.86)	0.71 (0.64, 0.77)	0.90 (0.84, 0.97)	0.0031	0.63 (0.61, 0.66)	0.81 (0.77, 0.84)	<.0001
M9	0.75 (0.66, 0.82)	0.67 (0.60, 0.74)	0.90 (0.85, 0.95)	<.0001	0.56 (0.48, 0.64)	0.75 (0.64, 0.87)	<.0001

- SS SumiShield 50 WG; BH: bottom half, TH: top half, C: ceiling; M: month
