# Supplementary material for: Efficacy of partial spraying of SumiShield, Fludora Fusion and Actellic against wild populations of Anopheles gambiae s.l. in experimental huts in Tiassalé, Côte d'Ivoire
Source: Sci Rep. 2023 Jul 13;13:11364. doi: 10.1038/s41598-023-38583-y (PMC10344869; doi:10.1038/s41598-023-38583-y)
Supplement: Supplementary file 7 — Supplementary Information 7. [file 41598_2023_38583_MOESM7_ESM.pdf]

Supp data 7: Insecticide sprayed residual life results using wall cone bioassay and wild collected resistant *An. gambiae* s.l. Tiassalé, per hut and sprayed surfaces

Month	SumiShield						Fludora Fusion						Actellic					
	SS Full		SS BH+C		SS TH+C		FF Full		FF BH+C		FF TH+C		AC Full		AC BH+C		AC TH+C	
	Wall	Ceiling	Wall	Ceiling	Wall	Ceiling	Wall	Ceiling	Wall	Ceiling	Wall	Ceiling	Wall	Ceiling	Wall	Ceiling	Wall	Ceiling
M0	100.0	100.0	100.0	100.0	100.0	100.0	100.0	100.0	100.0	100.0	100.0	100.0	100.0	100.0	100.0	100.0	100.0	100.0
M1	100.0	100.0	100.0	100.0	100.0	100.0	100.0	100.0	100.0	100.0	100.0	100.0	100.0	100.0	100.0	100.0	100.0	100.0
M2	100.0	100.0	100.0	100.0	100.0	100.0	100.0	100.0	100.0	100.0	100.0	100.0	100.0	100.0	100.0	100.0	100.0	100.0
M3	100.0	100.0	100.0	100.0	100.0	100.0	100.0	100.0	100.0	100.0	100.0	100.0	100.0	100.0	100.0	100.0	100.0	100.0
M4	100.0	100.0	100.0	100.0	100.0	100.0	100.0	100.0	100.0	100.0	100.0	100.0	100.0	100.0	100.0	100.0	100.0	100.0
M5	100.0	100.0	100.0	100.0	100.0	100.0	100.0	100.0	100.0	100.0	100.0	100.0	100.0	100.0	100.0	100.0	100.0	100.0
M6	100.0	100.0	100.0	100.0	100.0	100.0	100.0	100.0	100.0	100.0	100.0	100.0	97.4	100.0	97.5	100.0	100.0	100.0
M7	100.0	100.0	100.0	100.0	100.0	100.0	100.0	100.0	100.0	100.0	100.0	100.0	86.9	92.7	89.6	94.5	91.5	89.5
M8	95.6	89.2	92.7	91.8	93.5	96.8	100.0	100.0	100.0	100.0	100.0	100.0	69.8	73.7	73.5	79.8	67.9	77.9
M9	92.2	73.9	89.7	90.0	74.7	90.8	100.0	100.0	100.0	83.3	97.2	81.8	10.8	63.2	19.5	65.0	17.8	64.4

AC: Actellic 300 CS; FF: Fludora Fusion WP-SB; SS SumiShield 50 WG; BH: bottom half, TH: top half, C: ceiling, M: month
